# In Vitro Evaluation of Natural Sesquiterpene Lactones and Naphthoquinones Against Pancreatic Ductal Adenocarcinoma Cells

**DOI:** 10.3390/molecules31061014

**Published:** 2026-03-18

**Authors:** Nadia T. Mirakian, Rubén F. Iácono, Viviana B. Pulido, Matías A. Pibuel, Silvina L. Lompardía, Laura C. Laurella, Nicolás Pérez-Mauad, Cesar A. N. Catalán, Tomás Lombardo, Martín M. Ledesma, Adriana Carlucci, Valeria P. Sülsen, Daniela L. Papademetrio

**Affiliations:** 1 CONICET—Universidad de Buenos Aires, Instituto de Química y Metabolismo del Fármaco (IQUIMEFA), Ciudad Autónoma de Buenos Aires C1113AAD, Argentina; nmirakian@docente.ffyb.uba.ar (N.T.M.); c.laurella@docente.ffyb.uba.ar (L.C.L.); npmauad@ffyb.uba.ar (N.P.-M.); 2 Universidad de Buenos Aires, Facultad de Farmacia y Bioquímica, Cátedra de Inmunología, Ciudad Autónoma de Buenos Aires C1113AAD, Argentina; rubeniac@ffyb.uba.ar (R.F.I.); pibuelmatias@gmail.com (M.A.P.); slompardia@ffyb.uba.ar (S.L.L.); lomb.tomas@gmail.com (T.L.); 3 CONICET—Universidad de Buenos Aires, Instituto de Estudios de la Inmunidad Humoral (IDEHU), Ciudad Autónoma de Buenos Aires C1113AAD, Argentina; vivianabpulido@hotmail.com; 4 Universidad de Buenos Aires, Facultad de Farmacia y Bioquímica, Cátedra de Farmacognosia, Ciudad Autónoma de Buenos Aires C1113AAD, Argentina; 5 Facultad de Bioquímica, Química y Farmacia, Instituto de Química Orgánica, Universidad Nacional de Tucumán, San Miguel de Tucumán, Tucumán T4000INI, Argentina; cancatalan@gmail.com; 6 Unidad de Conocimiento Traslacional, Hospital de Alta Complejidad del Bicentenario Esteban Echeverría, Monte Grande B1842DOL, Argentina; mmledesma88@gmail.com; 7 CONICET—Hospital de Alta Complejidad del Bicentenario Esteban Echeverría, Centro de Investigaciones en Biomedicina Traslacional CIBiMeT, Monte Grande B1842DOL, Argentina; 8 Universidad de Buenos Aires, Facultad de Farmacia y Bioquímica, Departamento de Tecnología Farmacéutica, Instituto de Tecnología Farmacéutica u Biofarmacia (InTecFYB, UBA), Ciudad Autónoma de Buenos Aires C1113AAD, Argentina; adrianac@ffyb.uba.ar

**Keywords:** pancreatic ductal adenocarcinoma, sesquiterpene lactones, naphthoquinones, achillin, polymatin A, α,β-lapachone, lapachol

## Abstract

Pancreatic ductal adenocarcinoma (PDAC) is one of the most aggressive malignancies, highlighting the need to identify novel bioactive compounds with antitumor potential. Natural products constitute a valuable source of molecules with anticancer activity. In this study, we performed a comparative analysis of two classes of natural compounds—sesquiterpene lactones (achillin and polymatin A) and naphthoquinones (α, β-lapachone and lapachol)—in human PDAC cell lines on cell proliferation, metabolic activity and cell death induction and early mitochondrial alterations. Achillin showed limited antiproliferative, metabolic, and cytotoxic activity, whereas polymatin A exhibited activity in the micromolar range, yielding LC_50_ values of 16.11 ± 2.27 μM and 20.00 ± 1.90 μM for PANC-1 and MIAPaCa-2 cells, respectively. The naphtoquinones α- and β-lapachone effectively inhibited proliferation and metabolic activity and triggered cell death in both PDAC cell lines, with β-lapachone consistently displaying the highest activity with an LC_50_ of 4.00 ± 0.07 μM for PANC-1 cells and 3.89 ± 0.50 μM for MIAPaCa-2. Interestingly, achillin, polymatin A, α- and β-lapachone selectively induced cell death while sparing PBMCs. In contrast, lapachol showed weak activity, failing to achieve 50% inhibition or cell death within the tested concentration range and lacking tumor selectivity. Mechanistically, quinone derivatives promoted early mitochondrial superoxide modulation and membrane depolarization, consistent with a redox-active profile, whereas sesquiterpene lactones induced mitochondrial depolarization with limited mitochondrial superoxide overproduction, suggesting a distinct bioenergetic disruption phenotype. Overall, these findings highlight structure–activity relationships among natural compounds and support further investigation of achillin, polymatin A and α,β-lapachone as promising molecular scaffolds in PDAC research.

## 1. Introduction

Pancreatic ductal adenocarcinoma (PDAC) is one of the most aggressive and lethal malignancies worldwide, with a continuously rising global incidence [1,2]. The disease is characterized by late diagnosis, early metastasis, and high resistance to available treatments. Without therapy, metastatic PDAC confers a median survival of only 3–5 months, which increases to 6–10 months with standard treatments and to approximately 11–15 months after surgical resection in locally advanced cases [2]. Gemcitabine remains the conventional first-line therapy; however, it primarily induces cytostasis and yields only a modest extension of overall survival, increasing it by just 6.7 months on average [3]. These limitations highlight the urgent need to identify new therapeutic strategies capable of improving treatment response and patient outcomes.

Natural products constitute a source of structurally diverse bioactive molecules with proven relevance in oncology. More than half of all FDA-approved drugs between 1981 and 2019 are natural products or derivatives thereof, with particularly strong impact in the field of cancer therapy [4]. Within this chemical space, sesquiterpene lactones (STLs) and naphthoquinones have emerged as promising scaffolds due to their antiproliferative, pro-apoptotic, and redox-modulating properties.

**Figure 1:**
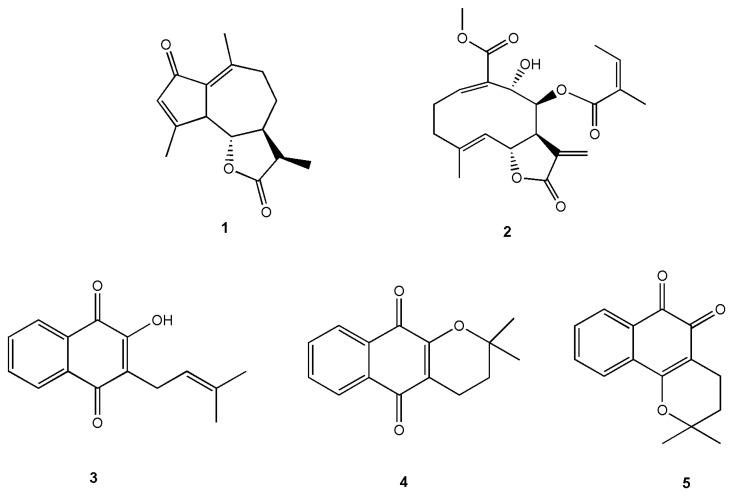
Chemical structure of the sesquiterpene lactones achillin (**1**) and polymatin A (**2**) and the naphthoquinones lapachol (**3**), α-lapachone (**4**) and β-lapachone (**5**).

Sesquiterpene lactones —widely distributed in the Asteraceae family—exhibit potent cytotoxic and antitumor activities [5,6,7,8]. Several representatives, such as parthenolide, arglabin, dehydrocostuslactone, and deoxyelephantopin, display activity against diverse cancer types, including breast cancer, leukemia, and pancreatic carcinoma [8]. Of particular relevance to this work are the STLs achillin (**1**) and polymatin A (**2**) (Figure 1), which were previously isolated from Argentine Asteraceae species and have been shown to exert cytotoxic effects on tumor cell lines [9,10].

Naphthoquinones comprise another class of natural compounds with significant biological potential. Among them, lapachol (**3**), α-lapachone (**4**), and β-lapachone (**5**) (Figure 1)—isolated from species of *Handroanthus* (lapacho)—have demonstrated noteworthy cytotoxicity and antitumor activity [11,12,13]. Lapachones have been widely studied for their ability to promote oxidative stress, disrupt mitochondrial and metabolic pathways, and induce programmed cell death in cancer cells. Their redox-active quinone core confers the capacity to trigger intracellular signaling alterations relevant to pancreatic cancer biology.

Sesquiterpene lactones and naphthoquinones were selected based on their activity on tumor cells and their potential as antitumor agents. Many compounds belonging to these groups have demonstrated the ability to interfere with redox-sensitive and stress-response pathways that are critical for PDAC survival. Despite their structural differences, these phytochemical groups may act through distinct molecular mechanisms, such as thiol alkylation and glutathione depletion in the case of STLs, or enzymatically driven redox cycling for naphthoquinones, while ultimately converging on metabolic and oxidative vulnerabilities characteristic of pancreatic cancer [14,15,16,17,18]. This duality provides a rationale for their comparative evaluation in the present study. Moreover, they may exert synergistic effects by acting on different biological targets.

Taken together, these precedents support the exploration of achillin, polymatin A, lapachol, α-lapachone, and β-lapachone as potential therapeutic agents for PDAC. Given the limited efficacy of current chemotherapeutics and the documented antitumor activities of these natural compounds, evaluating their effects on proliferation, metabolic activity, and cell death in human pancreatic cancer models represents a rational and timely research direction.

## 2. Results

### 2.1. Assessment of Cell Proliferation Inhibition

**Figure 2:**
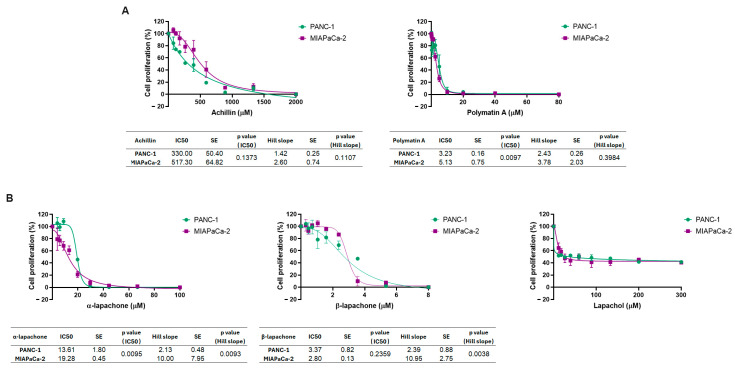
Antiproliferative effects of natural compounds on pancreatic cancer cell lines. The effects of (**A**) the sesquiterpene lactones achillin and polymatin A, and (**B**) the naphtoquinones α-lapachone, β-lapachone and lapachol on cell proliferation were evaluated by incorporation of BrdU in the pancreatic ductal adenocarcinoma cell lines PANC-1 and MIAPaCa-2. Dose–response curves were fitted using nonlinear regression. IC_50_ values, Hill slopes, standard errors, and statistical analyses are summarized in the tables shown below each graph, of six independent experiments.

The STLs achillin and polymatin A induced concentration-dependent inhibition of cell proliferation in both pancreatic cancer cell lines, PANC-1 and MIAPaCa-2 (Figure 2A). While both STLs fully suppressed cell proliferation in the two pancreatic cancer cell lines, they differed significantly in their anti-proliferative potency. Achillin showed IC_50_ values of 330.00 ± 50.40 µM in PANC-1 cells and 517.30 ± 64.82 µM in MIAPaCa-2 cells, without statistically significant differences in potency between cell lines. Polymatin A exhibited a markedly higher antiproliferative potency at lower concentrations, with IC_50_ values in the low micromolar range: 3.23 ± 0.16 µM for PANC-1 vs. 5.13 ± 0.75 µM for MIAPaCa-2, with PANC-1 cells significantly more sensitive than MIAPaCa-2 cells (*p* = 0.0097). For both achillin and polymatin A, nonsignificant differences were observed in the Hill slope values between the cell lines, indicating comparable dose–response curve steepness and suggesting similar underlying response dynamics.

Among the naphthoquinones, α-lapachone and β-lapachone significantly inhibited cell proliferation in both pancreatic cancer cell lines in a dose-dependent manner (Figure 2B). α-Lapachone achieved complete inhibition of cell proliferation in both cell lines, with IC_50_ values of 13.61 ± 1.80 µM in PANC-1 cells and 19.28 ± 0.46 µM in MIAPaCa-2 cells; the difference in potency between cell lines was statistically significant (*p* = 0.0095). In addition, significant difference in the Hill slope values was observed between PANC-1 and MIAPaCa-2 cells (*p* = 0.0093), indicating different dose–response curve behaviors.

β-Lapachone inhibited cell proliferation in both pancreatic cancer cell lines and exhibited greater antiproliferative potency than its α-isomer, with IC_50_ values of 3.37 ± 0.82 µM in PANC-1 and 2.80 ± 0.13 µM in MIAPaCa-2 cells. No statistically significant difference in IC_50_ values was observed between the two cell lines (*p* = 0.2359). Moreover, a significant difference in the Hill slope values between PANC-1 and MIAPaCa-2 cells was observed (*p* = 0.0038). By contrast, lapachol produced only a limited inhibitory effect on cell proliferation, failing to reach 50% inhibition within the tested concentrations and precluding IC_50_ estimation, thereby exhibiting markedly lower antiproliferative activity than α and β-lapachone and the STLs.

### 2.2. Effects of Natural Compounds on Cellular Redox Metabolic Activity

**Figure 3:**
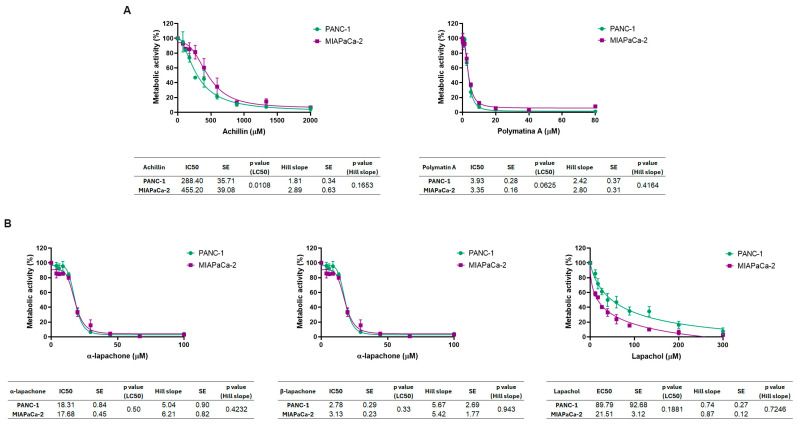
Effects of natural compounds on the metabolic activity of NAD(P)H-dependent reduction in XTT in pancreatic cancer cell lines. The effect of achillin and polymatin A (**A**) and α-lapachone, β-lapachone and lapachol (**B**) on cellular metabolic activity was evaluated using the XTT assay in the pancreatic ductal adenocarcinoma cell lines PANC-1 and MIAPaCa-2. Dose–response curves were fitted by nonlinear regression. IC_50_ values, Hill slopes, standard errors, and statistical analyses are summarized in the tables shown below each graph, of at least three independent experiments.

Achillin and polymatin A induced a concentration-dependent reduction in metabolic activity in both pancreatic cancer cell lines (Figure 3A). Achillin exhibited IC_50_ values of 288.40 ± 35.71 µM in PANC-1 cells and 455.20 ± 39.08 µM in MIAPaCa-2 cells, showing a significant difference in sensitivity between the two cell lines (*p* = 0.0108). Polymatin A demonstrated markedly higher potency, with IC_50_ values in the low micromolar range (3.930 ± 0.284 µM for PANC-1 and 3.350 ± 0.159 µM for MIAPaCa-2). However, no statistically significant differences in IC_50_ values between the two cell lines were observed (*p* = 0.0625). For both compounds, no significant differences were detected in the Hill slope values between PANC-1 and MIAPaCa-2 cells, indicating similar dose–response kinetics.

The naphthoquinones, α-lapachone and β-lapachone, triggered a pronounced, dose-dependent decrease in metabolic activity in both pancreatic cancer cell lines, achieving complete inhibition at the highest concentrations tested. α-Lapachone showed IC_50_ values of 18.31 ± 0.84 µM in PANC-1 cells and 17.68 ± 0.45 µM in MIAPaCa-2 cells, with no significant difference in potency (*p* = 0.4988). β-Lapachone exhibited higher antiproliferative activity, with IC_50_ values of 2.78 ± 0.29 µM for PANC-1 and 3.13 ± 0.23 µM for MIAPaCa-2 cells, also with no statistically significant difference between the two cell lines (*p* = 0.33) (Figure 3B). In contrast, lapachol only partially reduced metabolic activity and did not achieve 50% inhibition within the tested concentration range. Consequently, EC_50_ values were calculated instead of IC_50_, yielding EC_50_ values of 89.79 ± 92.68 µM for PANC-1 cells and 21.510 ± 3.122 µM for MIAPaCa-2 cells, with no statistically significant difference between the two cell lines (*p* = 0.1881).

Importantly, for all compounds tested, no significant differences in the Hill slope values were observed between PANC-1 and MIAPaCa-2 cells, suggesting similar response behavior.

### 2.3. Early Mitochondrial Alterations Induced by Natural Compounds

**Figure 4:**
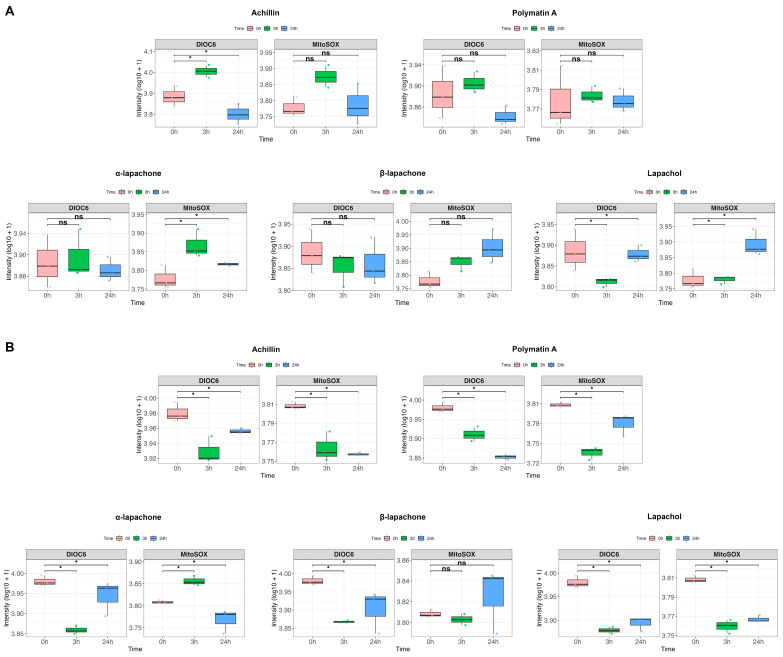
Effect of natural compounds on mitochondrial integrity. The effect of natural compounds on mitochondrial membrane potential was assessed by DIOC6 staining and the mitochondrial ROS production was evaluated by MitoSOX assay in PANC-1 (**A**) and MIAPaCa-2 (**B**) cells, at 3 and 24 h using the LD_50_ for each case. Box plots represent the median of three independent experiments. * *p* < 0.05, ns: not statistically significant.

To determine whether mitochondrial dysfunction precedes the cytotoxic effects detected at 72 h, mitochondrial membrane potential (ΔΨm) and mitochondrial superoxide production were evaluated at 3 h and 24 h (Figure 4).

In PANC-1 cells, STLs exerted limited mitochondrial effects. Polymatin A did not significantly modify ΔΨm, although a downward trend was observed at 24 h, and did not significantly alter mitochondrial ROS levels. Achillin induced only modest early changes in both parameters, without statistically significant alterations. In MIAPaCa-2 cells, mitochondrial responses to STLs were more evident. Polymatin A induced a significant early loss of ΔΨm at 3 h. Interestingly, mitochondrial superoxide levels decreased at 3 h and, although partially recovered at 24 h, remained significantly below basal values. Similarly, achillin produced early mitochondrial alterations without a marked increase in mitochondrial ROS production (Figure 4A).

In contrast, quinone derivatives displayed a clearer modulation of mitochondrial redox status. In PANC-1 cells, α-lapachone induced a significant increase in mitochondrial superoxide at 3 h. Although ROS levels declined at 24 h, they remained significantly higher than basal values, and these changes were accompanied by only minor alterations in ΔΨm. β-Lapachone induced a progressive increase in mitochondrial ROS, particularly evident at 24 h, together with a tendency toward mitochondrial depolarization. Lapachol produced early alterations in ΔΨm and modulated mitochondrial ROS levels over time. In MIAPaCa-2 cells, quinones induced more pronounced mitochondrial dysfunction. β-Lapachone triggered an early and marked loss of ΔΨm at 3 h, followed by increased mitochondrial ROS levels. α-Lapachone also reduced ΔΨm at early time points and increased mitochondrial superoxide production. Lapachol induced early mitochondrial depolarization without a clear increase in mitochondrial superoxide at 3 h (Figure 4B).

### 2.4. Cell Death Induction

**Figure 5:**
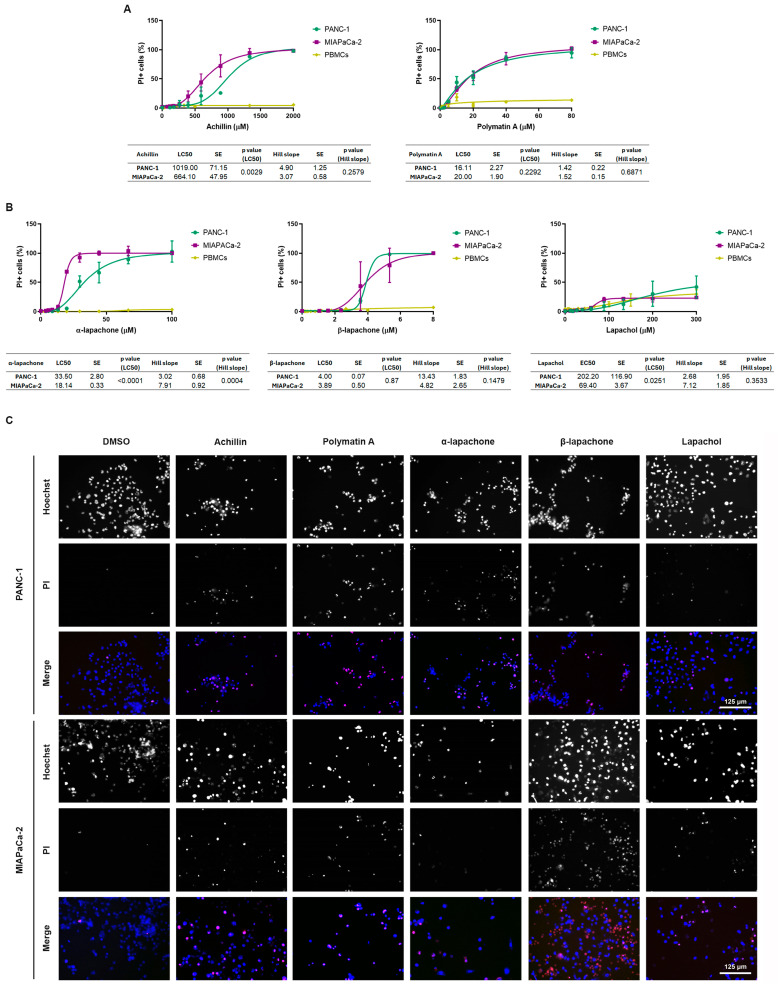
Induction of cell death by natural compounds in pancreatic cancer cell lines. The effect of achillin and polymatin A (**A**), and α-lapachone, β-lapachone and lapachol (**B**) on cellular viability was assessed by propidium iodide (PI) and Hoechst staining in the pancreatic ductal adenocarcinoma cell lines PANC-1 and MIAPaCa-2. Dose–response curves were fitted by nonlinear regression. LC_50_ values, Hill slopes, standard errors, and statistical analyses are summarized in the tables shown below each graph, of three independent experiments. (**C**) Representative images of PANC-1 and MIAPaCa-2 cells cultured in the presence of achillin (888 μM), polymatin A (20 µM), α-lapachone (44 μM), β-lapachone (5.33 μM) and lapachol (200 μM). DMSO was used as control vehicle.

Achillin and polymatin A induced dose-dependent cell death in both pancreatic cancer cell lines (Figure 5A), while exerting minimal cytotoxic effects on PBMCs across the tested concentration ranges. Achillin displayed moderate cytotoxic potency, with LC_50_ values of 1019.00 ± 71.15 µM for PANC-1 cells and 664.00 ± 47.95 µM for MIAPaCa-2 cells, indicating a significantly higher sensitivity of MIAPaCa-2 cells (*p* = 0.0029). Interestingly, polymatin A exhibited greater cytotoxic potency, with LC_50_ values of 16.11 ± 2.27 µM and 20.00 ± 1.89 µM for PANC-1 and MIAPaCa-2 cells, respectively, although no significant difference in LC_50_ values between the two cell lines was observed (*p* = 0.2292). For both compounds, PBMCs remained largely resistant, supporting a selective cytotoxic effect toward tumor cells. No significant differences in the Hill slope values were detected between PANC-1 and MIAPaCa-2 cells for either achillin or polymatin A, indicating similar dose–response kinetics of cell death induction.

Among the naphthoquinone derivatives (Figure 5B), α-lapachone and β-lapachone induced marked and concentration-dependent cell death in both pancreatic cancer cell lines, whereas PBMCs exhibited no sensitivity. α-Lapachone showed LC_50_ values of 33.50 ± 2.80 µM for PANC-1 cells and 18.14 ± 0.33 µM for MIAPaCa-2 cells, revealing a significantly higher susceptibility of MIAPaCa-2 cells (*p* < 0.0001). In addition, a significant difference in the Hill slope values was observed between the two cell lines (*p* = 0.0004), indicating distinct dose–response behaviors in terms of cell death induction.

β-Lapachone exhibited high cytotoxic potency, with comparable LC_50_ values in PANC-1 and MIAPaCa-2 cells (3.40 ± 0.07 µM and 3.89 ± 0.50 µM, respectively). No statistically significant difference in potency was observed between the cell lines (*p* = 0.8692). Similarly, no significant differences in the Hill slope values were detected, suggesting comparable response kinetics. In contrast, lapachol induced only partial cell death in both pancreatic cancer cell lines and failed to reach 50% cell death within the tested concentration range. PBMCs also exhibited low sensitivity to lapachol, with cell death levels comparable to those observed in tumor cells, indicating limited cytotoxic activity and a lack of tumor selectivity in comparison to the other naphthoquinones tested.

## 3. Discussion

The present study provides a comprehensive comparative analysis of two distinct types of natural compounds—STLs (achillin, polymatin A) and naphthoquinone derivatives (α,β-lapachone, lapachol)—in preclinical models of PDAC. The marked divergence in anti-proliferative and cytotoxic potencies offers critical insights into structure–activity relationships and mechanisms relevant to PDAC therapy. Importantly, the early mitochondrial alterations observed at 3–24 h provide additional mechanistic insight into these structure-dependent biological effects. Quinone derivatives (α-lapachone, β-lapachone and lapachol) consistently modulate superoxide production, supporting the notion that the quinone scaffold confers intrinsic redox-cycling capacity. Quinones are known to undergo enzymatic or non-enzymatic redox cycling, leading to superoxide generation and disturbance of intracellular redox homeostasis [18]. In our models, this was particularly evident for β-lapachone, which induced early mitochondrial ROS accumulation followed by loss of mitochondrial membrane potential, consistent with progressive mitochondrial dysfunction, resulting from NQO1-dependent futile redox cycling and sustained oxidative stress [19].

Achillin demonstrated slight anti-tumor activity, with IC_50_ values exceeding 295 µM in both PANC-1 and MIAPaCa-2 cells, translating to suboptimal metabolic inhibition (IC_50_ ~ 288–455 µM) and cytotoxicity (LC_50_ > 664 µM). This low potency aligns with the general requirement of high micromolar concentrations for many STLs to achieve significant NF-κB inhibition or oxidative stress [20]. However, achillin was able to enhance the chemosensitivity of Paclitaxel, overcoming resistance and increasing apoptosis in hepatocellular carcinoma cells [21]. In contrast, polymatin A exhibited low micromolar potency (IC_50_ ~ 3–5 µM), establishing it as the most potent lactone scaffold evaluated. This >100-fold enhancement likely reflects superior Michael acceptor reactivity of its α-methylene-γ-lactone moiety, absent in achillin, enabling efficient alkylation of cysteine residues in NF-κB subunits and other stress pathway proteins, a mechanism exploited by related STLs [22]. Thiol groups, particularly cysteine residues, together with free intracellular glutathione (GSH), represent the major targets of STLs. Interaction with these thiols interferes with GSH metabolism and disrupts intracellular redox homeostasis, leading to oxidative stress and activation of mitochondria-dependent apoptotic pathways. Notably, our mitochondrial data indicate that STLs (polymatin A and achillin) display a distinct mitochondrial phenotype compared with quinones. Although early mitochondrial depolarization was observed—particularly in MIAPaCa-2 cells—these compounds did not induce a marked increase in mitochondrial superoxide levels and, in some cases, even reduced MitoSOX signal at early time points. This suggests that mitochondrial protein modification or interference with redox-sensitive thiols may contribute to bioenergetic impairment without triggering overt superoxide overproduction. Most STLs exhibiting potent NF-κB inhibitory activity contain α-methylene-γ-lactone groups that act as alkylating agents, forming covalent adducts and inhibiting key enzymes and proteins. Structure–activity relationship studies have further demonstrated that saturation or substitution of the exo-methylene group markedly reduces cytotoxic and antitumor activity, whereas additional alkylating elements enhance potency, underscoring the central role of the α-methylene-γ-lactone structure in STLs bioactivity [15,16]. Notably, structural modification of the α-methylene-γ-butyrolactone moiety results in a significant reduction in cytotoxic activity, further supporting the central role of this pharmacophore in the anticancer activity of STLs [23]. Regarding achillin, previous studies have demonstrated its chemosensitizing properties, enhancing the cytotoxic and apoptotic effects of paclitaxel [21].

The observed selectivity index, preserving PBMC viability while inducing tumor cell death (LC_50_ ~ 16–20 µM), mirrors the tumor-selective toxicity reported for other DNA polymerase-inhibiting natural products, such as Helenalin and bis (helenalinyl) malonate [24]. Importantly, this mechanism is not restricted to these compounds, as other STLs also inhibit DNA polymerase and thymidylate synthase in tumor cells, thereby suppressing nuclear DNA synthesis [25]. The differential sensitivity between PANC-1 and MIAPaCa-2 cells for polymatin A (*p* = 0.0097) suggests lineage-specific expression of its molecular target(s). Given that PANC-1 harbors mutant p53 while MIAPaCa-2 is p53-null, the enhanced polymatin A activity in PANC-1 may be associated with exploitation of DNA repair deficiencies, analogous to the synthetic lethality observed with β-lapachone in XRCC1-deficient PDA cells [26]. It is worth mentioning that the related compound polymatin B has been evaluated in tumor cell lines, including MIAPaCa-2 cells. Polymatin B differs from polymatin A by the presence of an acetoxy group at C-9 instead of a hydroxyl group. This compound exhibited an IC_50_ value of 3.7 ± 0.2 µM, as determined using the MTT assay. This activity is comparable to that observed for polymatin A, suggesting that the acetoxy substitution at C-9 does not play a critical role in modulating metabolic activity. In PMBC cells, polymatin B showed an IC_50_ > 20 µM, indicating a selectivity of action, consistent with the behavior observed for polymatin A [27].

The isomeric α- and β-lapachones displayed greatly different efficacies that underscore the critical role of bioactivation. β-Lapachone emerged as exceptionally potent (IC_50_ ~ 2.8–3.4 µM), with similar activity in both PDAC lines, reflecting uniform NQO1 expression and enzymatic activity in these models. This NQO1-dependent futile redox cycle culminates in PARP1 hyperactivation, rapid NAD^+^/ATP depletion, and programmed necrosis—a mechanism extensively validated in PDAC xenografts and phase I trials (ARQ761) [28]. The early increase in mitochondrial ROS and subsequent depolarization observed in our study is fully consistent with this redox-driven bioenergetic collapse. Importantly, these mitochondrial alterations precede overt cell death at 72 h, supporting their role as initiating events in the cytotoxic cascade. Our cytotoxicity data (LC_50_ ~ 3.9–4.0 µM) directly correlate with the metabolic catastrophe threshold reported for NQO1^+^ cancers, where NAD^+^ pools collapse below 10% within hours [28].

α-Lapachone was approximately 5–6-fold less potent than β-lapachone, exhibiting IC_50_ values in the range of 13.6–19.3 µM and showing a statistically significant greater sensitivity in PANC-1 cells compared with MIAPaCa-2 cells (*p* = 0.0095). This reduced activity aligns with the inability of α-lapachone to undergo efficient NQO1-mediated bioactivation; instead, it likely operates via alternative mechanisms such as direct topoisomerase I/II inhibition and ROS generation [29]. The differential Hill slope (*p* = 0.0093) between the cell lines suggests distinct engagement of these alternative pathways, possibly reflecting variations in topoisomerase expression or redox buffering capacity, as previously documented for AsPC-1 vs. MIAPaCa-2 cells, where enhanced antioxidant capacity is associated with reduced β-lapachone potency [28]. The superior performance of β-lapachone suggests that the 2,2-dimethyl-2H-pyran ring may be essential for optimal NQO1 substrate recognition and redox cycling [30]. The parent naphthoquinone, lapachol, failed to achieve a measurable IC_50_ (EC_50_ ~ 21–90 µM), confirming that the absence of the dimethyl substituents abrogates both NQO1-dependent and -independent anti-tumor effects at therapeutic concentrations. This recapitulates findings that lapachol lacks the potency required for monotherapy, despite its historical use and moderate PKM2 inhibition [31]. Its minimal PBMC toxicity, however, suggests a favorable safety profile that could be leveraged for combinatorial strategies. Both 1,2- and 1,4-naphthoquinones undergo enzymatic reduction to semiquinone and hydroquinone intermediates, leading to increased generation of reactive oxygen species (ROS). The resulting hydroquinone species can also act as DNA-alkylating agents, causing irreversible DNA damage and ultimately triggering apoptotic cell death in naphthoquinone-treated cells. In addition, 1,2-naphthoquinones have been shown to covalently trap topoisomerase II, thereby impairing enzyme function [32]. In the case of β-lapachone, its antitumor activity has been primarily attributed to NQO1-mediated redox cycling, which drives sustained reactive oxygen species (ROS) production and ultimately suppresses tumor cell growth. In addition to this redox-dependent mechanism, β-lapachone has been reported to inhibit a broad range of oncogenic enzymes, including topoisomerase I, topoisomerase II, IDO1, telomerase, and Hsp90, further contributing to its antitumor efficacy [33]. Recent evidence indicates that β-lapachone exerts significant antitumor effects in NQO1-positive cancer cells, a phenotype characteristic of most solid tumors, by inducing immunogenic cell death (ICD) and enhancing tumor immunogenicity [34].

The therapeutic index, defined by the window between tumor cytotoxicity (LC_50_) and PBMC viability, was most favorable for β-lapachone and polymatin A. Both agents preserved immune cell integrity while executing profound tumor cell death, a prerequisite for immunocompetent PDAC models where chemotherapy-induced lymphopenia exacerbates immunosuppression. The lack of PBMC toxicity for β-lapachone corroborates its established tumor-selective mechanism, the NQO1 bioactivatable drug (NQO1-BAD) paradigm, which spares NQO1-deficient normal tissues [26].

For polymatin A, the mechanistic basis of selectivity remains to be fully elucidated. This selectivity may involve an increase in vulnerability of PDAC cells to oxidative stress due to oncogenic KRAS-driven metabolic reprogramming. Similar selective lethality has been reported for the naphtoquinone shikonin and the STLs parthenolide and micheliolide in glycolysis-dependent tumors [15,16]. Future studies should evaluate polymatin A in combination with β-lapachone or GLS1 inhibitors to exploit metabolic synthetic lethality, as demonstrated for β-lapachone combinations that enhance PARP1 hyperactivation beyond monotherapy thresholds [35].

The biological activities of STLs are mainly attributed to the presence of the α-methylene-γ-lactone moiety. Nevertheless, additional structural features may influence their potency and selectivity. In particular, the α,β-unsaturated system can react with sulfhydryl groups in enzymes and other proteins, potentially leading to toxic effects. Contact dermatitis is among the most commonly reported adverse reactions associated with STLs [36,37]. Regarding naphthoquinones, hemolytic effects have been reported for some compounds of this class [38]. In the case of lapachol, adverse effects such as anemia, nausea, and vomiting have also been described [39]. In this context, the design and synthesis of structural derivatives have emerged as promising strategies to improve the selectivity of these compounds while maintaining or enhancing their biological activity. Additionally, the development of targeted delivery systems, such as nanodelivery, may further increase selectivity and reduce systemic side effects.

### Final Considerations and Future Directions

While our in vitro data establish structure–activity relationships hierarchies, our findings also reveal scaffold-dependent early mitochondrial phenotypes that may underlie the differential cytotoxic responses observed at later time points. However, in vivo validation remains essential. The superior potency of β-lapachone must be balanced against well-documented clinical limitations, including dose-limiting anemia, short plasma half-life, and suboptimal intratumoral bioavailability. Nanoformulation strategies, such as liposomal or albumin-bound β-lapachone, may improve pharmacokinetics while preserving NQO1-dependent activation, which could help overcome these limitations. Conversely, polymatin A’s novel scaffold warrants evaluation in xenograft and genetically engineered mouse models of PDAC. Given its distinct bioenergetic disruption profile without marked mitochondrial superoxide overproduction, further studies should determine whether this phenotype translates into improved tolerability or complementary activity in vivo. Such studies are required to confirm its promising therapeutic window and identify predictive biomarkers of response, including p53 status or NF-κB activation signatures, or markers of metabolic and mitochondrial vulnerability.

## 4. Materials and Methods

### 4.1. Reagents

Propidium iodide (PI) (P417), XTT and phenazine methosulfate (PMS) (Phenazine methosulfate P9625) were purchased from Sigma-Aldrich (St. Louis, MO, USA). HOECHST 33342 (cat H21492), DMEM (cat 12800017), l-glutamine (35050061), streptomycin and penicillin (cat 15140122) were purchased from Invitrogen (Waltham, MA, USA). DIOC6 (CAS 53213-82-4) (Sigma-Aldrich, St. Louis, MO, USA) and MitoSOX (cat M36008; Invitrogen, Waltham, MA, USA) were kindly provided by Dr. Daniel Gonzalez Maglio. SFB was purchased from Internegocios (Mercedes, Buenos Aires, Argentina). BrdU, monoclonal mouse anti-BrdU (317902) antibody, and goat anti-mouse HRP (405306) secondary antibody were purchased from Biolegend (San Diego, CA, USA). Lapachol (CAS 84-79-7), α-lapachone (CAS 4707-33-9) and β-lapachone (CAS 4707-32-8) were purchased from BLD Pharmatech Ltd. (Shanghai, China). Achillin (CAS 5956-04-7) was obtained from the aerial parts of *Artemisia copa* (Asteraceae) (LIL S/N) as previously described by Mercado et al. and Catalán et al. [40,41]. Polymatin A (CAS 72023-29-1)was isolated from *Smallanthus macrocyphus* aerial parts (LIL 607375) as previously reported by Coll Aráoz et al. and De Pedro et al. [42,43].

### 4.2. Cell Cultures

MIAPaCa-2 (clone CRL-1420) and PANC-1 (clone CRL-1469) cells were obtained from the American Type Culture Collection (ATCC) (Manassas, VA, USA). The cells were cultured in DMEM containing 10% heat-inactivated FBS, 2 mM L-glutamine, 20 mM HEPES buffer, 100 IU/mL penicillin, and 150 µg/mL streptomycin at 37 °C in a humidified incubator with 5% CO_2_ and tested for Mycoplasma every three months via PCR (Abcam cat# ab289834). Cells at fewer than 20 passages were used for the experiments described. Cell viability was determined by trypan blue exclusion.

### 4.3. Cell Treatments

For all assays, cells were seeded 48 h before treatment. Cells were treated with 0–1000 μM of achillin, 0–80 μM of polymatin A, 0–100 μM of α-lapachone, 0–8 μM of β-lapachone or 0–300 μM of lapachol. All compounds were tested using serial 2/3 dilutions, except Polymatin A, which was evaluated using serial 1/2 dilutions. Untreated and vehicle control cultures were also included. All incubations were performed at 37 °C in a 5% CO_2_ atmosphere for 72 h.

### 4.4. Cell Proliferation Assay

For the evaluation of cell proliferation, 3 × 10^3^ cells/well were seeded in 96-well plates and treated for 70 h as described in 4.3. Then, BrdU was added at a final concentration of 20 µM and cells were incubated for 2 h more. After this time, the supernatant was removed, the cells were washed, fixed with PFA 4% for 20 min and permeabilized with HCl 2N. The reaction was neutralized with sodium tetraborate (0.1 M; pH 9) and the endogenous peroxidase activity was blocked with 3% H_2_O_2_ in methanol for 30 min at room temperature. After that, cells were blocked with FBS 2% O.N. and incubated with mouse anti-BrdU antibody (1/1000) O.N. at 4 °C. Finally, HRP conjugated anti-mouse antibody was added (1/2000) and incubated for 2 h at room temperature. The assay was revealed with TMB and the reaction was stopped after 10 min with H_2_SO_4_ 4N. Absorbance was read at 450 nm and 620 nm using a microplate reader (Multiscan Ex, Absorbance Microplate Reader, Thermo Electron Corporation, Shanghai, China). Cell proliferation was calculated as:

[Abs (treated)450 − Abs (treated)620/Abs (untreated)450 − Abs (untreated)620] × 100.

### 4.5. Metabolic Activity Assay

For studying the metabolic activity of the compounds, XTT assay was employed: 3 × 10^3^ cells/well were seeded in 96-well plates and treated as described in 4.3. for 70 h. After treatment, culture medium was discarded, and 100 µL of an XTT solution (1 mg/mL) containing PMS (7.5 µg/mL) was added to each well. Cells were incubated for 2 additional hours at 37 °C in a 5% CO_2_ atmosphere. The absorbance (Abs) was read at 450 nm and 620 nm using a microplate reader (Multiscan Ex, Absorbance Microplate Reader, Thermo Electron Corporation, China). Cell viability was calculated as:

[Abs (treated)450 − Abs (treated)620/Abs (untreated)450 − Abs (untreated)620] × 100.

### 4.6. Assessment of Mitochondrial Membrane Potential and Superoxide Production

Mitochondrial membrane potential (ΔΨm) and mitochondrial superoxide production were evaluated using the fluorescent dyes DIOC6 (3,3′-dihexyloxacarbocyanine iodide, Sigma-Aldrich, St. Louis, MO, USA) and MitoSOX™ Red (Cat. M36008, Invitrogen, Waltham, MA, USA), respectively. MIAPaCa-2 and PANC-1 cells were seeded at 5 × 10^4^ cells/well in 24-well plates and allowed to adhere for 24 h. Cells were then treated with the indicated compounds (achillin, polymatin, α-lapachone, β-lapachone, and lapachol) at LD50 concentrations for 3 and 24 h. After compound incubation, cells were washed once with DPBS and incubated with the appropriate dyes at a final concentration of 20 nM for DIOC6 and 2.5 μM for MitoSOX. CCCP (carbonyl cyanide m-chlorophenyl hydrazone) was used as a positive control for ΔΨm dissipation. Following 30 min of incubation, cells were washed with 1 mL DPBS and detached using trypsin. Cell suspensions were transferred to 1.5 mL tubes and kept on ice until analysis. Flow cytometry was performed in a Sysmex-Cyflow equipment (IMEX-ANM, Buenoas Aires, Argentina). Raw cytometry events were imported from FCS files using flowCore 2.16.0 (Bioconductor, Seattle, WA, USA; Vancouver, BC, Canada), and event-level matrices were converted to data frames for downstream analysis with dplyr, tidyr, and tibble (tidyverse).

### 4.7. Cell Death Assay

The induction of cell death by the compounds was also evaluated. For this purpose, 3 × 10^3^ cells/well were seeded in 96-well plates and treated for 72 h. Cells were then stained with Hoechst 33342 to a final concentration of 1.33 μM for 15 min. PI was added to a final concentration of 0.6 μg/mL for 15 min. Stained cells were acquired on an EVOS M7000 (ThermoFisher) and image analysis was performed using FIJI (v1.54.r) software.

### 4.8. Statistical Analysis

Dose–response relationships were analyzed using GraphPad Prism (version 8; GraphPad Software, San Diego, CA, USA). Experimental data were fitted by nonlinear regression using a four-parameter logistic model (4PL) with variable slope. Half-maximal inhibitory (IC_50_), lethal (LC_50_), or effective (EC_50_) concentrations were calculated depending on the experimental endpoint evaluated (proliferation, metabolic activity, or cell death). Statistical comparisons between dose–response curves were carried out using the extra sum-of-squares F test, to determine whether the curves best describe the datasets by comparing goodness of fit. For flow cytometry results, inferential analyses were conducted at the tube/replicate level, summarizing each tube by the median DIOC6 and median MitoSOX signal. A nonparametric analysis, timepoint differences (0 h, 3 h, 24 h) were tested for each drug × cell line × marker combination using Kruskal–Wallis on tube-level medians, followed by Dunn’s post hoc comparisons versus basal using FSA (dunnTest), and displayed as boxplots with significance annotations in ggplot2 4.0.1. Differences were considered statistically significant when *p* < 0.05.

### 4.9. Chemical Structure Generation

Chemical structures of the compounds were generated using ChemOffice 2016 (ChemDraw Professional 2016, PerkinElmer, Waltham, MA, USA) and verified using the PubChem database.

## 5. Conclusions

Taken together, our results reveal a clear hierarchy of biological activity among the compounds tested. Polymatin A and β-lapachone combine high potency with consistent dose–response kinetics and favorable tumor selectivity, whereas achillin displays moderate activity and lapachol exhibits limited and non-selective effects. Importantly, the concordance between antiproliferative, metabolic, and cytotoxic outcomes strengthens the biological relevance of these findings and supports the use of integrated functional assays to identify compounds with genuine antitumoral potential.

## Data Availability

The original contributions presented in the study are included in the article; further inquiries can be directed to the corresponding authors.

## Abbreviations

The following abbreviations are used in this manuscript:

ATCC	American Type Culture Collection
BrdU	5-bromo-2′-deoxyuridine
DMEM	Dulbecco’s Modified Eagle Medium
DMSO	Dimethyl sulfoxide
EC_50_	Half-maximal effective concentration
FBS	Fetal bovine serum
HRP	Horseradish peroxidase
IC_50_	Half-maximal inhibitory concentration
LC_50_	Half-maximal lethal concentration
MTT	3-(4,5-dimethylthiazol-2-yl)-2,5-diphenyltetrazolium bromide
NF-κB	Nuclear factor kappa B
NQO1	NAD(P)H:quinone oxidoreductase 1
PARP1	Poly(ADP-ribose) polymerase 1
PBMCs	Peripheral blood mononuclear cells
PBS	Phosphate-buffered saline
PDAC	Pancreatic ductal adenocarcinoma
PI	Propidium iodide
ROS	Reactive oxygen species
STLs	Sesquiterpene lactones
XTT	Sodium 3′-[1-(phenylaminocarbonyl)-3,4-tetrazolium]-bis(4-methoxy-6-nitro)benzene sulfonic acid hydrate
